# Cardiopulmonary Exercise Testing Distinguishes between Post-COVID-19 as a Dysfunctional Syndrome and Organ Pathologies

**DOI:** 10.3390/ijerph191811421

**Published:** 2022-09-10

**Authors:** Johannes Kersten, Luis Hoyo, Alexander Wolf, Elina Hüll, Samuel Nunn, Marijana Tadic, Dominik Scharnbeck, Wolfgang Rottbauer, Dominik Buckert

**Affiliations:** Department for Internal Medicine II, University of Ulm, 89081 Ulm, Germany

**Keywords:** cardiopulmonary exercise testing (CPET), COVID-19, dysfunctional breathing, deconditioning, long COVID-19, post-COVID-19

## Abstract

(1) Background: Dyspnea is one of the most frequent symptoms among post-COVID-19 patients. Cardiopulmonary exercise testing (CPET) is key to a differential diagnosis of dyspnea. This study aimed to describe and classify patterns of cardiopulmonary dysfunction in post-COVID-19 patients, using CPET. (2) Methods: A total of 143 symptomatic post-COVID-19 patients were included in the study. All patients underwent CPET, including oxygen consumption, slope of minute ventilation to CO_2_ production, and capillary blood gas testing, and were evaluated for signs of limitation by two experienced examiners. In total, 120 patients reached a satisfactory level of exertion and were included in further analyses. (3) Results: Using CPET, cardiovascular diseases such as venous thromboembolism or ischemic and nonischemic heart disease were identified as either cardiac (4.2%) or pulmonary vascular (5.8%) limitations. Some patients also exhibited dysfunctional states, such as deconditioning (15.8%) or pulmonary mechanical limitation (9.2%), mostly resulting from dysfunctional breathing patterns. Most (65%) patients showed no signs of limitation. (4) Conclusions: CPET can identify patients with distinct limitation patterns, and potentially guide further therapy and rehabilitation. Dysfunctional breathing and deconditioning are crucial factors for the evaluation of post-COVID-19 patients, as they can differentiate these dysfunctional syndromes from organic diseases. This highlights the importance of dynamic (as opposed to static) investigations in the post-COVID-19 context.

## 1. Introduction

COVID-19 is known to affect several organs and organ systems, besides the respiratory tract. Long-term symptoms months after COVID-19 are reported independently of the initial course of the disease, and include fatigue, dyspnea, chest pressure, olfactory dysfunction, and headaches [1,2,3]. This syndrome of persistent symptoms after COVID-19 is called post-COVID-19, long COVID-19, or post-acute sequelae of COVID-19. In view of the substantial number of patients affected, post-COVID-19 results in a considerable medical and socioeconomic burden.

Various explanations for post-COVID-19, including immunological changes, persistence of the virus or viral components, and behavioral, inflammatory, or thrombotic processes, have been proposed [4,5]. The correct diagnostic workup, especially regarding the allocation of resources, given the lack of evidence-based therapy, is also part of the discussion. Since some diagnostics such as cardiovascular magnetic resonance imaging, neuropsychological examinations, or comprehensive interdisciplinary diagnostic battery are expensive or time-intensive, not every patient recovering from COVID-19 has access to them. Thus, a stepwise diagnostic approach seems reasonable [6]. Since cardiopulmonary exercise testing (CPET) is a standard diagnostic for differentiating among diverse causes of dyspnea and exercise intolerance [7], its use seems reasonable with post-COVID-19 patients. Previous studies evaluating cardiopulmonary function in broad collectives showed that physical performance in these patients was reduced compared with healthy subjects but was similar to patients with unexplained dyspnea from the pre-COVID-19 era [8,9]. In contrast to the evaluation of absolute values in the heterogeneous cohorts of post-COVID-19 patients, it seems reasonable to identify patterns of impairment. In this way, it is possible to exclude differential diagnoses on the one hand, and to make a specific diagnosis of “post-COVID syndrome” on the other. The aim of this study is to describe patterns of cardiopulmonary dysfunction of post-COVID-19 patients using CPET.

## 2. Materials and Methods

### 2.1. Study Design and Patient Enrollment

Patients presenting to the post-COVID-19 unit of our University Tertiary Care Center were diagnosed using a previously described stepwise diagnostic approach [6]. They had to have new-onset and persistent symptoms following COVID-19 disease or shortly thereafter. This had to be at least 3 months previous. In short, the first step included an anamnestic evaluation, a 12-lead electrocardiography, laboratory tests, a transthoracic echocardiography, and a body plethysmography, including testing of the diffusing capacity for carbon monoxide (DLCO), capillary blood gas testing, and a six-minute walk test (6-MWT). For the second step—CPET evaluation—the inclusion criteria were established as otherwise unexplainable alterations in the initial diagnostics, including (1) a left ventricular ejection fraction below 55%; (2) increased levels of high-sensitivity troponin T (>15 ng/L) or N-terminal brain natriuretic peptides (>250 pg/mL); (3) reduced (<80% of the target) total lung capacity, forced expiratory volume in one second, Tiffeneau–Pinelli index (FEV1/FVC ratio), or DLCO; (4) relevant alterations in capillary blood gas testing; and (5) a distance of less than 450 m covered in the 6-MWT or ≥7 points on the Borg scale for dyspnea or exhaustion at the end of the test. Some patients with a high symptom burden but otherwise normal results in the initial diagnostics also underwent CPET. A high symptom burden included subjective impairments that occurred in association with COVID-19 disease or shortly thereafter and resulted in persistent sick leave or the inability to participate in social life.

All patients provided written informed consent. This study was approved by the Ethics Committee of the University of Ulm (approval number 406/20) and conducted in accordance with the Declaration of Helsinki. Neither the patients nor the public were involved in the design, conduct, reporting, or dissemination plans of this research.

**Figure 1:**
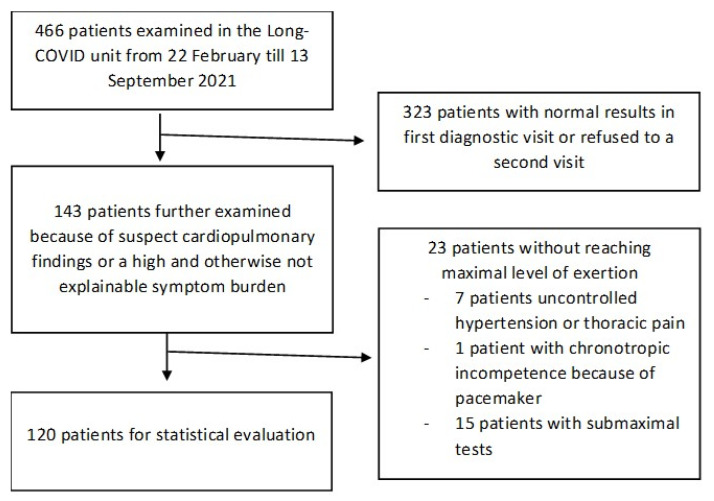
Flowchart of patient enrollment.

A total of 466 patients were prospectively examined in our post-COVID-19 unit and underwent the baseline diagnostics described above. A total of 108 patients underwent CPET due to suspect findings in the initial diagnostics, and these constituted the cardiopulmonary suspect group. They included 22 patients with a reduced left ventricular ejection fraction, 12 patients with elevated cardiac biomarkers, 65 patients with reduced values in body plethysmography (including DLCO), 4 patients with alterations in capillary blood gas testing, and 38 patients with short 6-MWT distances or high Borg scale scores. Some patients exhibited more than one sign. A total of 35 patients with ongoing symptoms but no pathological findings in the initial diagnostics also underwent CPET. Out of the 23 patients who did not achieve a satisfactory level of exertion, 15 suffered severe respiratory distress without reaching the objective criteria for adequate exertion. In total, 120 patients remained for statistical analysis. The patient enrollment procedure is illustrated in Figure 1.

### 2.2. Cardiopulmonary Exercise Testing

CPET was performed using a bicycle ergometer with the patient in a half-lying position, with a resting phase prior to exercise and a recovery phase afterward. Capillary blood gas measurements were performed at rest, around the anaerobic threshold (AT), at maximum exercise, and in the recovery phase. The patients were monitored using electrocardiograms, noninvasive blood pressure measurements, pulse oximetry, and ventilatory gas measurements, using a commercially available diagnostic station (Vyntus CPX; Vyaire Medical GmbH, Hoechberg, Germany), and the results were presented in nine-panel plots. CPET was considered to indicate a satisfactory level of exertion if one or more of the following criteria were met: (1) a respiratory exchange ratio (RER: the ratio of carbon dioxide exhaling [VCO_2_] to oxygen uptake [VO_2_]) of 1.10 or higher; (2) maximum lactate of 5 mmol/L or more; or (3) a breathing frequency of 50 breaths/min. Rarer conditions, such as desaturation (partial pressure of oxygen below 55 mmHg during exercise), were considered to indicate that an individual had reached maximum exhaustion (adopted after [10,11,12]). AT was defined as the crossing point of VO_2_ and VCO_2_ (RER = 1.00), which has been shown to correlate well with the lactate-AT and to have good reproducibility and comparability [13,14,15,16]. The target VO_2_ was calculated using the Wasserman equation. The VO_2_ AT was assumed to be normal if it was more than 40% of the target VO_2_. 

The CPET results were evaluated for signs of limitation by two experienced examiners by consensus and in accordance with state-of-the-art criteria [7,10,11,12,17,18]. Limitation was diagnosed in the case of a workload below the predicted value and peak VO_2_ below 85% of the target. Additional factors, such as an early increase in breathing frequency (over 50 breaths/min), a highly elevated minute ventilation/carbon dioxide production (VE/VCO_2_) or minute ventilation/oxygen uptake (VE/VO_2_) slope with no signs of metabolic or cardiac exertion, a dynamic increase in end-expiratory lung volume, or a chaotic/dysfunctional breathing pattern [19,20], were considered signs of pulmonary–mechanic limitation. Cardiac limitation was defined as a reduced VO_2_ pulse, an early plateau of the VO_2_ pulse curve, chronotropic incompetence, or a low increase in VO_2_ (<8 mL/W) with otherwise normal ventilatory parameters. Pulmonary–vascular limitation was diagnosed in the case of a high (and potentially increasing) alveolar-to-arterial oxygen partial pressure difference (AaDO_2_), a significant increase in the VE/VCO_2_ slope (>35), and a reduction in end-tidal CO_2_ without significant increments during exercise. Deconditioning was diagnosed in cases of peak VO_2_ below 85% of the target, a potentially slight decrease in VO_2_ pulse, and early tachycardia and drained breathing reserve. Other ventilatory parameters and blood gases had to be normal, and pulmonary–mechanic, cardiac, or pulmonary–vascular limitations were excluded. 

### 2.3. Statistics

IBM SPSS Statistics 26 (IBM, Armonk, NY, USA) was used for the statistical analysis. Continuous variables were expressed as means ± standard deviations, and categorical values were expressed as numbers and percentages. All data were normally distributed under graphical analysis or when subjected to the Kolmogorov–Smirnov test. The study is descriptive in most parts. Comparative analyses were performed using ANOVA or a *t*-test, as appropriate. Statistical significance was assumed for a *p*-value < 0.05.

## 3. Results

Our study cohort consisted mostly of women (61.7%) with a mean age of 49.7 ± 15.2 years. CPET examinations took place 227 ± 114 days after the initial COVID-19 disease. The initial COVID-19 disease showed mostly a mild-to-moderate course, with hospitalized patients accounting for only 15.8%. The most common symptoms at the initial presentation were dyspnea, fatigue, memory and concentration disorders, and thoracic pain or pressure (Table 1). The CPET results for the entire cohort are shown in Table 2. As can be seen, the achieved values for the collective are on average the normative for workload and peak VO_2_. Time from negativity to CPET performance had no significant effect on either peak VO_2_, nor VO_2_AT in a logistic regression analysis (*p* = 0.645 and *p* = 0.202). A comparison of patients with and without previous cardiovascular disease showed no significant difference regarding maximal workload (1.8 ± 0.7 W/kg vs. 1.9 ± 0.6 W/kg, *p* = 0.358), peakVO_2_ (22.0 ± 7.8 mL/min/kg vs. 24.8 ± 7.0 mL/min/kg, *p* = 0.227), or VO_2_AT (15.8 ± 6.1 mL/min/kg vs. 17.4 ± 5.6 mL/min/kg, *p* = 0.391). Thus, a significant influence of known cardiovascular disease on the other results seems unlikely. Similar results were found for pre-existing and known lung disease and cancer (each *p* > 0.05).

**Figure 2:**
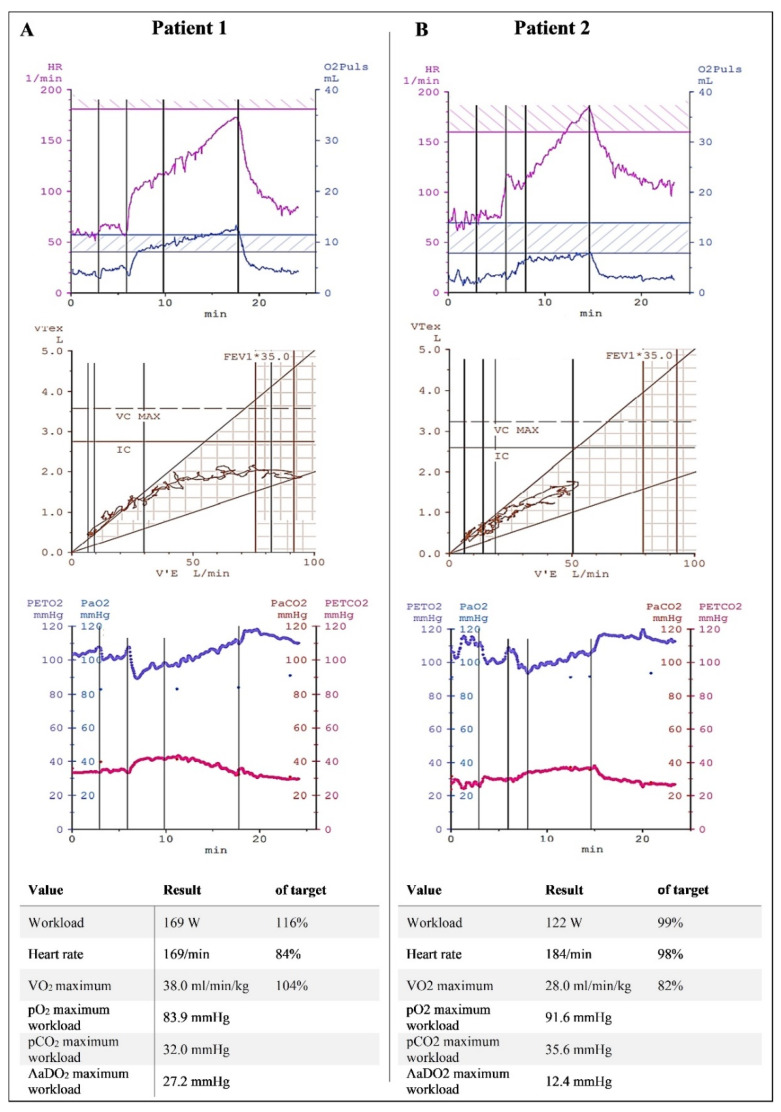
Cardiopulmonary exercise testing of a patient with no limitation (**A**) and a patient with deconditioning (**B**). The patient with no limitation showed a good and steady increase in heart rate and VO_2_ pulse (top panel), with normal ventilatory volumes (middle panel) and expiratory gases (bottom panel). The workload and capillary blood gases were within normal ranges. The patient with deconditioning also showed a normal heart rate, normal ventilatory volumes, and normal blood gases, but significantly reduced VO_2_ and a VO_2_ pulse under the target corridor. (FEV1*35 is the product of one second capacity and 35 to estimate the maximum voluntary ventilation.)

Normal CPET with no signs of cardiopulmonary limitation was the most frequent finding (78/120 or 65% of patients), with a maximum workload of 157 ± 53 W (129.0 ± 28.9% of the target) and peak VO_2_ of 27.0 ± 7.0 mL/min/kg (106.9 ± 15.4% of the target). However, a considerable number of patients (19/120; 15.8%) exhibited deconditioning. These patients showed a nearly normal workload (123 ± 26 W; 89 ± 8.7% of the target) but reduced peak VO_2_ (21.8 ± 5.5 mL/min/kg; 78.7 ± 4.1% of the target), with no other pathological findings related to breathing patterns or respiratory gases. Examples of a patient with no limitation and a patient with deconditioning are shown in Figure 2.

**Figure 3:**
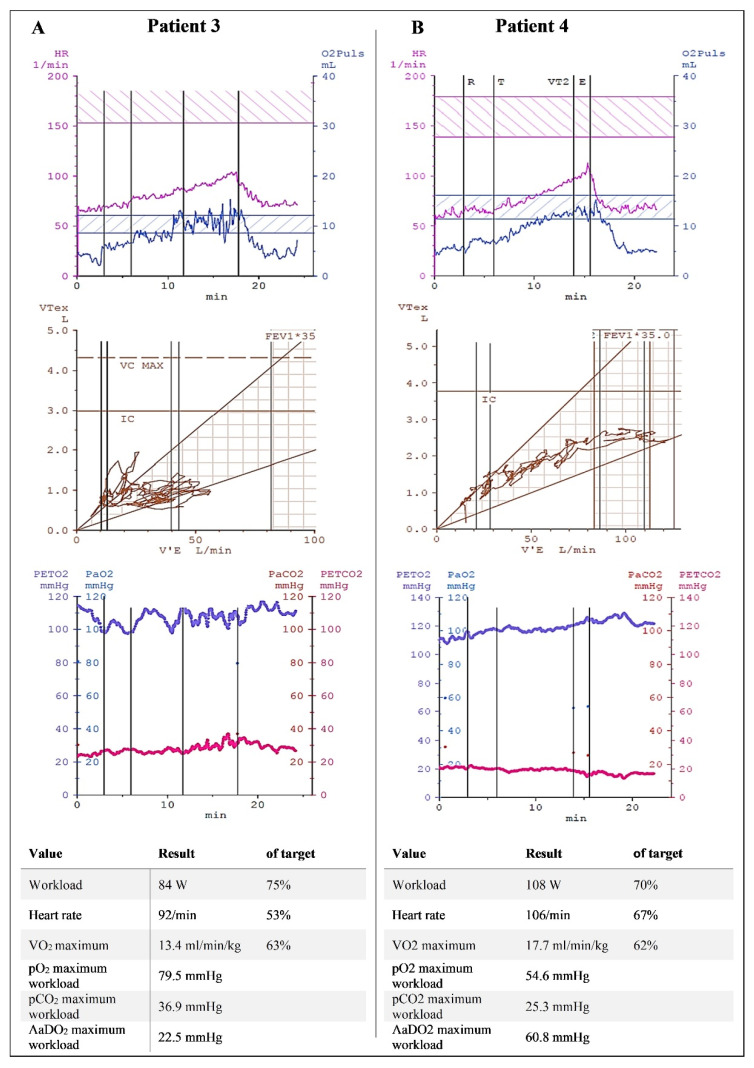
Cardiopulmonary exercise testing of a patient with dysfunctional/chaotic breathing (**A**) and a patient with pulmonary–vascular limitation (**B**). The patient with dysfunctional breathing was characterized by erratic and abrupt changes in respiratory rate (middle), with subsequent increased dead space and early termination at only 75% of the predicted workload. The patient with pulmonary–vascular limitation had normal ventilatory volumes (middle) with low expiratory carbon dioxide (red line, bottom panel). The alveolar-to-arterial oxygen partial pressure difference was extremely high (60.8 mmHg). (FEV1*35 is the product of one second capacity and 35 to estimate the maximum voluntary ventilation.)

Eleven (9.2%) patients with pulmonary–mechanical limitation formed another group. This group included one patient with dynamic hyperinflation and an otherwise restrictive or chaotic breathing pattern characterized by a constantly changing respiratory frequency and depth (Figure 3). Patients with dysfunctional breathing had no cardiocirculatory abnormalities or gas exchange problems. They were limited only by their breathing patterns.

**Figure 4:**
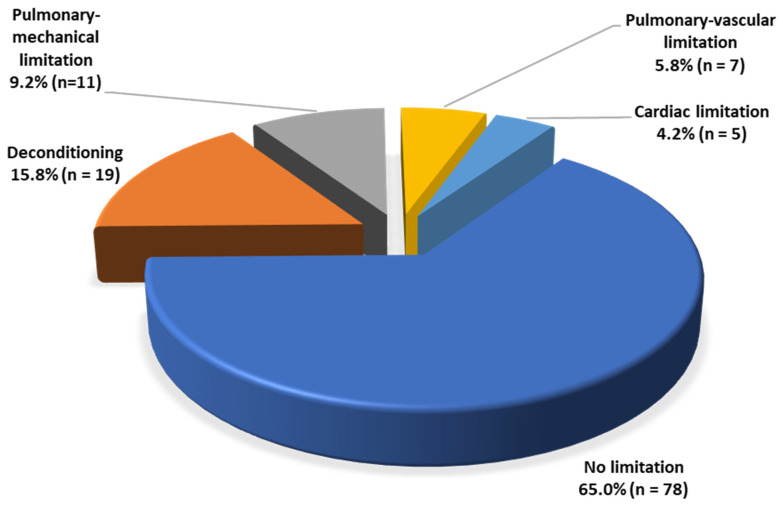
Classification of cardiopulmonary limitations of all patients reaching the maximal level of exertion (n = 120).

Few patients showed signs of cardiac (5/120; 4.2%) or pulmonary–vascular (7/120; 5.8%) limitation. Both groups showed a slight reduction in maximal workload, with a mean of 135 ± 9 W (86.8% ± 16.1 of the target) for patients with cardiac limitation and 127 ± 43 W (89.6% ± 26.8 of the target) for patients with pulmonary–vascular limitation. Peak VO_2_ was also reduced for both groups: 16.7 ± 2.6 mL/min/kg (74.6% ± 7.6 of the target) and 21.0 ± 7.6 mL/min/kg (80.3% ± 17.9 of the target), respectively. The distribution of limitations in our cohort is shown in Figure 4.

Patients were referred for further diagnostics, such as left- and right-heart catheterization, bronchoscopy, or chest CT scans, depending on clinical needs and pretest probability. Venous thromboembolism and ischemic or nonischemic heart disease were predominant in the cardiac and pulmonary–vascular limitation groups. Except for one case of sarcoidosis, no structural cardiac or pulmonary disease was detected in the pulmonary–mechanical limitation group or deconditioning group, indicating functional rather than structural issues. 

A comparison of patients hospitalized for initial COVID-19 with non-hospitalized and oligo-/asymptomatic courses, found lower peak VO_2_, and lower pO_2_ and higher AaDO_2_ during exercise. Other values of CPET including workload were not significantly different. Detailed results can be found in Table 3.

## 4. Discussion

The main findings of this study are as follows: (1) CPET showed high diagnostic value with regard to the diagnostic workup of cardiopulmonary symptoms in the post-COVID-19 context; (2) there were a number of potentially pre-existing and exacerbated or newly developed cardiopulmonary organic diseases; (3) there were dysfunctional patterns, including deconditioning and dysfunctional or chaotic breathing, in the absence of clear structural organic diseases. (Our suggestion would be “functional” post-COVID-19 syndrome.)

CPET is the gold standard for the evaluation and verification of dyspnea. However, few studies have used CPET for the evaluation of post-COVID-19 patients. Cassar et al. [8] examined 26 patients two to three months after COVID-19, and found a significant reduction in VO_2_ and an improvement in values (although still reduced) after six months (18.0 [16.1–27.9] mL/kg/min vs. 20.5 [17.5–26.1] mL/kg/min; *p* < 0.001). This cohort consisted of previously hospitalized patients with a high proportion of intensive care unit admissions (37%). Conversely, our cohort consisted mostly of mild-to-moderate initial COVID-19 cases and generally showed normal workload and VO_2_ values. Post-COVID-19 patients with prior hospitalization due to COVID-19 have been shown to have more pathological findings [21]. Previously hospitalized patients also had worse peak VO_2_ values in our cohort.

In our study, 23 patients (16.1%) did not achieve a satisfactory level of exertion. Submaximal exertion has been observed previously in post-COVID-19 cases. A severe initial disease course and a recent case of COVID-19 disease were identified as risk factors for exercise intolerance [22].

Another study using CPET suggested that deconditioning was a major factor in functional impairment [23]. Patients with deconditioning also constituted a considerable proportion of our cohort. This can be attributed to presumed deconditioning caused by both the initial disease itself and changed lifestyle habits during the pandemic [24]. However, myopathic changes must also be considered [25].

Dysfunctional breathing has also been reported in post-COVID-19 patients [19]. Given that there is no universal definition of erratic breathing, and because selection bias cannot be ruled out in any relevant study, the real prevalence of dysfunctional breathing in daily life remains unknown. We assume a significant influence of this dysfunction on symptom development in post-COVID-19 patients. Patients fulfilling the criteria for this pattern in CPET may represent just the tip of the iceberg. Standardized diagnostic criteria and larger cohorts would be helpful in characterizing dysfunctional breathing. Erratic breathing and other frequently occurring syndromes, such as postural tachycardia, sleep disorders, and fatigue, have been suspected to be related to the brainstem [26]. Increased neuroinflammation or even neuroinvasion of the brainstem by SARS-CoV-2 has been discovered in autopsy studies [27]. Other cohort studies using 18F PET imaging have found hypometabolism in post-COVID-19 patients and a correlation with the symptom burden [28,29]. Interestingly, a similar pattern has been found in patients with myalgic encephalomyelitis/chronic fatigue syndrome (ME/CFS) [30]. This lends weight to the assumption that post-COVID-19 is not a new entity, but rather a condition that has been described in other post-inflammatory syndromes. Mancini et al. found that a high proportion (42%) of patients fulfilled the diagnostic criteria for ME/CFS, with a significant overlap with chronic hyperventilation and dysfunctional breathing [19].

Given the high number of post-COVID-19 patients and their heterogeneity, a simple and generalizable cause of the symptoms seems unlikely. There is an overlap with other diseases, as also seen in our cohort. We found a sizable proportion of patients with (presumably) pre-existing diseases, such as coronary artery disease or dilative cardiomyopathy, or conditions induced by the initial COVID-19 disease, such as pulmonary embolism. As far as we could ascertain, almost all patients with cardiac or pulmonary–vascular limitation and none with other limitations had a cardiovascular disease that could explain the symptoms leading to their presentation at our post-COVID-19 unit. It is therefore reasonable to distinguish between cardiovascular disease and cardiovascular symptoms, in the absence of an organic correlate, in the context of post-COVID, as suggested by the American College of Cardiology [31]. Our findings suggest that CPET could be essential for distinguishing between patients with cardiovascular symptoms only, and those with cardiovascular disease. The exclusion of cardiopulmonary performance limitation is equally important in this patient population, as it can trigger further noncardiac differential diagnoses and provide further recommendations for further rehabilitation.

The categorization of limitations using CPET also provides an opportunity for further therapy and training recommendations. Whereas in patients with cardiac and pulmonary vascular performance limitations the focus is on diagnostics, for the reasons mentioned above, for the remaining patients, physical exercise is useful [32,33]. CPET has been recommended before to identify training corridors and provide exercise prescriptions [33,34]. Due to a possible overlap with ME/CFS [19], patients with dysfunctional breathing in particular must be advised to start with easy training and increase slowly [35,36]. Overtraining and overexertion may be counterproductive in this setting. In patients with pulmonary mechanical limitation or dysfunctional breathing, activation of the diaphragm under professional guidance may be useful [37].

In most studies of post-COVID-19 and CPET, pediatric patients are excluded. In addition, in our study, the minimum age for inclusion was 18 years. A case report by Buonsenso et al. reported a 14-year-old girl with ongoing symptoms 7 months after COVID-19 [38]. Static and imaging studies (spirometry, cardiac magnetic resonance imaging, chest CT) did not show any pathology. CPET revealed pulmonary vascular limitation, which the authors attributed to persistent inflammation and microvascular dysfunction of the pulmonary vessels. Further studies including pediatric patients with post-COVID-19 are needed.

## 5. Limitations

As with other post-COVID-19 investigations, our study is susceptible to selection bias. Nevertheless, our cohort is, to our knowledge, the largest CPET cohort of post-COVID-19 patients. The study’s relatively small sample size and single-center design are clear limitations, as is the lack of a control group without prior SARS-CoV-2 infection. There are no systematic data on cardiopulmonary exercise capacity in our patients before COVID-19 disease, so a causal relationship between the observed conditions and infection can only be presumed. For greater generalizability, especially in terms of differentiation between organic and purely functional changes, standardized diagnostics for all patient groups using chest CT and/or left- and right-heart catheterization would be desirable. Lastly, our study also lacks a matched cohort of probands functioning as controls who do not have a history of COVID-19 disease.

## 6. Conclusions

For those patients with suspect findings in basic cardiopulmonary diagnostics especially, CPET can provide insights into potential underlying pathological changes. The mean cardiopulmonary exercise capacity in our cohort was normal, but CPET can also detect different patterns of limitation. It thus enables the differentiation between exacerbated pre-existing cardiovascular disease and dysfunctional states, such as deconditioning or dysfunctional breathing, which are potential targets for distinct rehabilitation. It is therefore reasonable to add CPET to the standard diagnostic workup of patients presenting with ongoing cardiovascular symptoms after SARS-CoV-2 infection.

## Figures and Tables

**Table 1:** Patient characteristics (n = 120).

Characteristic	Value
**Age (years), mean ± SD**	49.7 ± 15.2
**Women, n (%)**	74 (61.7)
**Body mass index (kg/m^2^), mean ± SD**	25.4 ± 4.3
**Patient history, n (%)**	
Cardiac diseases	10 (8.3)
Pulmonary diseases	15 (12.5) (asthma bronchial, 14 (11.7))
Malignant diseases	2 (1.7)
**COVID-19 history, n (%)**	
Oligosymptomatic/asymptomatic course	10 (8.3)
Hospitalization	19 (15.8)
Invasive ventilation	6 (5.0)
Therapy with corticosteroids	12 (10.0)
Therapy with antibiotics	14 (11.7)
**Cardiovascular risk profile, n (%)**	
Arterial hypertension	30 (25.0)
Diabetes mellitus type I	2 (1.7)
Diabetes mellitus type II	6 (5.0)
Dyslipidemia	68 (56.7)
Current/past smoking	29 (24.2)
**Post-COVID-19 symptoms, n (%)**	
Thoracic pain/pressure	32 (26.7)
Dyspnea	82 (68.3)
Anosmia/ageusia	10 (8.3)
Headaches	10 (8.3)
Sleep disorders	16 (13.4)
Exhaustion/fatigue	71 (59.2)
Memory and concentration disorders	41 (34.5)

**Table 2:** Cardiopulmonary exercise testing of the patients (n = 120).

Measurement	Value	Predicted/Norm *
Workload (W)	144 ± 50	132 ± 46
Workload per bodyweight (W/kg)	1.9 ± 0.6	1.8 ± 0.6
Heart rate (min^−1^)	147 ± 25	170 ± 15
Expiratory volume (L/min)	77.0 ± 22.7	91.5 ± 16.7
Peak VO_2_ (mL/min/kg)	24.6 ± 7.1	26.2 ± 7.5
VO_2_AT (mL/min/kg)	17.3 ± 5.7	10.5 ± 3.0
VE/VCO_2_	33.1 ± 6.2	<35
VO_2_ pulse (mL/beat)	12.8 ± 3.7	≥9
VO_2_ work rate (mL/W)	13.1 ± 1.5	≥10
pO_2_ baseline (mmHg)	77.2 ± 7.8	
pO_2_ maximum workload (mmHg)	81.9 ± 10.4	
pCO_2_ baseline (mmHg)	36.0 ± 3.5	
pCO_2_ maximum workload (mmHg)	33.8 ± 3.9	
AaDO_2_ baseline (mmHg)	16.3 ± 8.1	
AaDO_2_ maximum workload (mmHg)	24.1 ± 10.0	
Maximum lactate (mmol/L)	7.3 ± 2.6	

AaDO_2_: alveolar-to-arterial oxygen partial pressure difference; pCO_2_: partial pressure of carbon dioxide; Peak VO_2_: peak oxygen consumption; pO_2_: partial pressure of oxygen; VE: minute ventilation; VCO_2_: carbon dioxide exhaling/production; VO_2_AT: oxygen consumption at the anaerobic threshold. * Adopted from [7,10,11,12,17,18]. Values are expressed as mean ± SD (calculated based on individual thresholds) or as absolute thresholds.

**Table 3:** Cardiopulmonary exercise testing depending on initial disease severity, divided into hospitalized patients, non-hospitalized patients, and oligo-/asymptomatic courses.

Characteristic/Measurement	Hospitalized (n = 19)	Non-Hospitalized (n = 91)	Oligo-/Asymptomatic Course (n = 10)	*p*-Value
Workload (W)	144 ± 36	141 ± 47	178 ± 78	0.074
Workload per bodyweight (W/kg)	1.7 ± 0.5	1.9 ± 0.6	2.2 ± 0.8	0.104
Heart rate (min^−1^)	134 ± 28	150 ± 25	149 ± 20	0.053
Expiratory volume (L/min)	80.4 ± 21.1	75.7 ± 23.1	82.2 ± 23.1	0.538
Peak VO_2_ (mL/min/kg)	20.8 ± 5.4	25.0 ± 6.9	27.8 ± 9.4	0.022
VO_2_AT (mL/min/kg)	16.3 ± 3.6	17.1 ± 5.8	20.8 ± 7.2	0.109
VE/VCO_2_	33.8 ± 8.7	33.1 ± 5.7	31.1 ± 5.2	0.511
VO_2_ pulse (mL/beat)	13.9 ± 3.4	12.3 ± 3.4	15.0 ± 5.4	0.034
VO_2_ work rate (mL/W)	12.7 ± 1.2	13.2 ± 1.5	12.8 ± 1.2	0.297
pO_2_ baseline (mmHg)	72.4 ± 8.3	78.1 ± 7.7	76.6 ± 6.7	0.057
pO_2_ maximum workload (mmHg)	74.2 ± 12.6	84.0 ± 9.1	77.3 ± 9.0	<0.001
pCO_2_ baseline (mmHg)	36.2 ± 3.6	35.8 ± 3.6	37.1 ± 2.9	0.529
pCO_2_ maximum workload (mmHg)	35.8 ± 5.1	33.2 ± 3.4	35.7 ± 4.2	0.007
AaDO_2_ baseline (mmHg)	20.6 ± 7.8	15.4 ± 7.9	15.6 ± 8.1	0.037
AaDO_2_ maximum workload (mmHg)	30.1 ± 13.3	22.6 ± 9.0	26.1 ± 7.3	0.008
Maximum lactate (mmol/L)	7.6 ± 2.9	7.3 ± 2.6	7.1 ± 1.9	0.861

AaDO_2_: alveolar-to-arterial oxygen partial pressure difference; pCO_2_: partial pressure of carbon dioxide; Peak VO_2_: peak oxygen consumption; pO_2_: partial pressure of oxygen; VE: minute ventilation; VCO_2_: carbon dioxide exhaling/production; VO_2_AT: oxygen consumption at the anaerobic threshold.

## Data Availability

The datasets used and/or analyzed during the current study are available from the corresponding author on reasonable request.
